# Heparan Sulfate Proteoglycans (HSPGs) and Their Degradation in Health and Disease

**DOI:** 10.3390/biom15111597

**Published:** 2025-11-14

**Authors:** Nicola Greco, Valentina Masola, Maurizio Onisto

**Affiliations:** 1 Department of Engineering for Innovative Medicine, University of Verona, Strada le Grazie 15, 37134 Verona, Italy; nicola.greco@studenti.unipd.it; 2 Department of Biomedical Sciences, University of Padova, Viale G. Colombo 3, 35121 Padova, Italy; valentina.masola@unipd.it

**Keywords:** heparan sulfate proteoglycans (HSPGs), heparanase (HPSE), extracellular matrix (ECM)

## Abstract

Heparan sulfate proteoglycans (HSPGs) are essential constituents of the extracellular matrix (ECM) and cell surface, orchestrating a wide range of biological processes, such as cell adhesion, migration, proliferation, and intercellular communication. Through their highly sulfated glycosaminoglycan chains, HSPGs serve as crucial modulators of bioavailability and signaling of growth factors, cytokines, and chemokines, thereby influencing tissue homeostasis. Their dynamic remodeling is mediated by numerous enzymes, with heparanase (HPSE) playing a predominant role as the only known human endo-β-D-glucuronidase that specifically cleaves heparan sulfate chains. Beyond its well-documented enzymatic activity in ECM degradation and the release of HS-bound molecules, HPSE also exerts non-enzymatic functions that regulate intracellular signaling cascades, transcriptional programs, and immune cell behavior. Dysregulated HPSE expression or activity has been implicated in various pathological conditions, including fibrosis, chronic inflammation, cancer progression, angiogenesis, metastasis, and immune evasion, positioning this enzyme as a pivotal driver of ECM plasticity in both health and disease. This review provides an updated overview of HSPG biosynthesis, structure, localization, and functional roles, emphasizing the activity of HPSE and its impact on tissue remodeling and disease pathogenesis. We further explored its involvement in the hallmark processes of cancer, the inflammatory tumor microenvironment, and its contribution to fibrosis. Finally, we summarize current therapeutic strategies targeting HPSE, outlining their potential to restore ECM homeostasis and counteract HPSE-driven pathological mechanisms. A deeper understanding of the HSPG/HPSE axis may pave the way for innovative therapeutic interventions in cancer, inflammatory disorders, and fibrotic diseases.

## 1. Introduction

The ECM is a complex and dynamic 3D network of molecules that provides structural and functional support to the surrounding cells. It includes structural proteins such as collagen, elastin, fibronectin, proteoglycans, and hyaluronic acid, which act as regulators of tissue fluids and signaling molecules. Moreover, the ECM coordinates not only cell growth, proliferation, and migration, but also tissue development, homeostasis, and wound healing by modulating intracellular signaling pathways [1]. In the last decade, the complexity of ECM composition and architecture has been deciphered thanks to proteomic and transcriptomic approaches that have led to a greater understanding of the so-called “matrisome”, i.e., the ensemble of ECM proteins and associated factors [2]. Each tissue is characterized by a specific composition of the matrisome that is generated in the early embryonic stages, and each cell type has a precise repertoire of ECM receptors that mediate cell-ECM interactions [3]. The matrisome is composed of “core” ECM proteins, which include proteoglycans, collagens, and multi-adhesive proteins, and matrisome-associated factors, such as secreted cytokines, growth factors, and remodeling enzymes [3,4]. In this review, we focused our attention on proteoglycans, with particular attention to heparan sulfate proteoglycans and their enzymatic degradation by heparanase under normal and pathological conditions.

## 2. Proteoglycans (PGs)

Proteoglycans (PGs) are large glycoproteins characterized by a specific core protein to which one or more polysaccharide chains are covalently attached. These polysaccharide chains, known as glycosaminoglycans (GAGs), are long, unbranched, and consist of repeating disaccharide units composed of hexosamine (N-acetyl-D-glucosamine, GlcNAc, or N-acetylgalactosamine, GalNAc), uronic acid (D-glucuronic GlcA or L-iduronic acid, IdoA), and/or galactose. During their assembly in the Golgi apparatus, these disaccharide units undergo sulfation at various positions, which imparts a high negative charge to GAGs. The significant structural diversity of proteoglycans arises from (1) the vast array of core proteins identified, (2) the number of GAG chains linked to the core protein, and (3) the type of GAG, which is classified based on its sugar components and modification patterns into chondroitin sulfate, dermatan sulfate, keratan sulfate, and heparan sulfate/heparin. Additionally, proteoglycans can have different types of GAGs on the same core protein, forming what is known as a hybrid proteoglycan [1,5]. Proteoglycans can be categorized based on their glycosaminoglycan (GAG) side chains and the nature of their core proteins. They are generally grouped into three main families: small leucine-rich proteoglycans (e.g., decorin and biglycan), large aggregating proteoglycans (e.g., aggrecan and versican), and cell-surface proteoglycans (e.g., syndecan-4 and glypican-1) [6].

## 3. Heparan Sulfate Proteoglycan: Biosynthesis and Structure

Heparan sulfate proteoglycans (HSPGs) are a class of complex molecules found in the ECM and cell surface [7]. They play an important role in various physiological processes, especially in cell–cell communication, cell adhesion, and regulation of different cell signaling pathways. HSPGs share a common structural feature with the proteoglycan family; however, they are characterized by the presence of highly sulfated GAG side chains [8]. For this reason, HSPGs have a strong negative charge that confers the ability to interact with a broad range of molecules, including growth factors, cytokines, and other ligands. Indeed, they are essential for regulating the bioavailability, distribution, and activity of different signaling molecules, influencing various cellular pathways [9].

Heparan sulfate proteoglycans (HSPGs) represent a heterogeneous class of molecules composed of different protein cores covalently conjugated to heparan sulfate (HS) chains of variable length. HS is formed by repeating disaccharide units composed of either glucuronic acid (GlcA) or its epimer iduronic acid (IdoA), together with N-acetylglucosamine (GlcNAc) or N-sulfoglucosamine (GlcNS). The disaccharides are linked through α(1 → 4) or β(1 → 4) glycosidic bonds, while the inter-disaccharide connections are consistently of the α(1 → 4) type [1,7,10]. The protein core is synthesized in the endoplasmic reticulum, whereas HSPG biosynthesis proceeds in the Golgi apparatus, the site of HS chain polymerization and modification. Polymerization is initiated at a tetrasaccharide linkage region composed of xylose, galactose, and glucuronic acid, which is attached to specific serine residues of the core protein. The elongation of HS chains is mediated by exostosin-1 and -2 (EXT1 and EXT2), two type II transmembrane glycosyltransferases localized in the Golgi apparatus, and further refined through the sequential action of specialized enzymes. The first modification involves the N-deacetylation and N-sulfation of GlcNAc, catalyzed by N-deacetylase/N-sulfotransferases (NDSTs), followed by the C5-epimerization of GlcA into IdoA. Subsequent modifications include 2-O-sulfation of IdoA and 6-O-sulfation of GlcNAc. This step is of particular importance in cancer signaling and progression, as it is closely linked to the functional properties of HSPGs [10,11]. Depending on the sulfation pattern, HS can be organized into low-sulfated regions and highly sulfated domains [12,13]. Moreover, the presence of non-sulfated regions composed of GlcA and GlcNAc plays a critical role in defining the spectrum of molecules capable of interacting with HSPGs [14,15].

## 4. Heparan Sulfate Proteoglycan: Localization and Function

**Figure 1:**
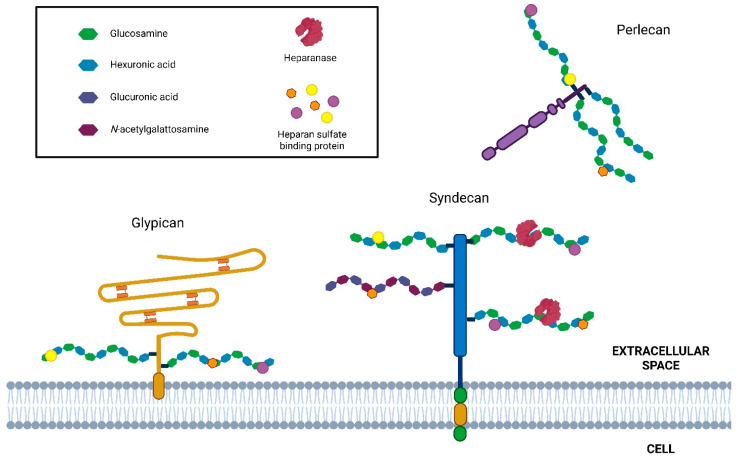
Schematic representation of HSPG and HPSE enzymatic activity. Principal cell surface (syndecan and glypican) and basement membrane-associated (perlecan) HSPG. HS chains (highlighted in black box) are cleaved by heparanase, producing HS fragments (5–10 kDa). In addition, this activity promotes the ECM remodeling and the release of many HS-linked molecules, including growth factors, chemokines, enzymes, lipoproteins, and plasma proteins.

Cells possess a relatively limited repertoire of HSPGs (approximately 17), which can be classified into three main categories based on their localization: membrane-associated HSPGs, including syndecans and glycosylphosphatidylinositol (GPI)-anchored proteoglycans such as glypicans; secreted extracellular matrix HSPGs, such as agrin, perlecan, and type XVIII collagen; and secretory vesicle proteoglycans, exemplified by serglycin [9,12] (Figure 1).

## 5. Basement Membrane-Associated HS-Proteoglycans

Basement membrane-associated HSPGs are important components of the extracellular scaffold, but also act as modulators of signaling pathways and morphogen gradients through interactions with regulatory and signaling factors [6]. This group of HSPGs comprises perlecan, agrin, and collagen XVIII. Perlecan, localized at the basement membrane, is characterized by a multidomain protein core and three glycosaminoglycan (GAG) chains, primarily HS, attached at its N-terminus. Beyond its structural role, perlecan interacts with various extracellular matrix (ECM) components, growth factors, and membrane proteins such as integrins and tyrosine kinase receptors, thereby influencing multiple biological processes. Notably, perlecan contributes to vascularization and tumor angiogenesis, where its HS chains act as reservoirs for vascular endothelial growth factor (VEGF), platelet-derived growth factor (PDGF), transforming growth factor-β (TGF-β), and members of the fibroblast growth factor (FGF) family [16,17]. Agrin is a multimodular HSPG containing three HS chains present at the basement membrane level. Agrin is abundant in the synaptic region, which plays an important role as an organizer of the neuromuscular junction at the postsynaptic membrane due to the high affinity of its N-terminus to laminin in the basal membrane and C-terminal domain to low-density lipoprotein-like receptor 4 (LRP4) in skeletal muscles [18]. Collagen XVIII is a ubiquitous component prevalent in the basement membrane of vascular and endothelial cells. It is a structurally complex homotrimer organized in a triple helix and presents three HS chains and an endostatin domain located at the C-terminus [19]. The last member is the testican, recently called SPOCK, which is a modular HSPG with 2–5 HS chains associated with the C-terminal domain. Three members of this family are expressed in the central nervous system and are principally involved in neuronal development [6].

## 6. Cell Surface Proteoglycans

Cell surface proteoglycans (PGs) are predominantly heparan sulfate proteoglycans (HSPGs), which are anchored to the plasma membrane either through a transmembrane core protein or a glycosylphosphatidylinositol (GPI) linkage. These molecules fulfill diverse functions, acting as adhesion mediators, endocytic receptors, and co-receptors, thereby modulating processes such as signal transduction, cell adhesion, and motility. The two principal families of cell surface proteoglycans are syndecans and glypicans [20]. The syndecan family consists of four members, known as syndecan 1–4 (SDC 1–4). These transmembrane proteins feature three domains: a small intracellular C-terminal domain, transmembrane region, and N-terminal extracellular domain, where the heparan sulfate (HS) chains are attached distally to the plasma membrane. Additionally, SDC-1 and -3 have proximal chondroitin sulfate chains. The transmembrane domain is a highly conserved sequence, whereas the ectodomain is the most variable region [9,21]. Membrane HSPGs can activate receptors on the same cell (in cis) or neighboring cells (in trans), facilitating cell–cell communication. A notable example is SDC-1, which enhances the binding of basic fibroblast growth factor-2 (FGF-2) and activation of FGF-2 receptor-1 (FGFR1). Specifically, the released HS fragments bound to FGF-2 are strong activators of FGFR1 [22]. Glypicans are highly conserved proteins characterized by a cysteine-rich extracellular protein core attached to the plasma membrane through a GPI anchor. This family includes six glypicans (1–6), each with distinct biological properties related to cellular responses to various growth factors and morphogens [23]. A unique member of the cell surface HSPG family is the transforming growth factor (TGF-β) type III receptor, which is also known as betaglycan. It can bind to TGF-β, facilitating its binding to the type II receptor via the protein core and to FGF-2 via HS [24]. Membrane proteoglycans undergo post-translational regulation through ectodomain shedding, which generates soluble HSPGs able to redistribute HS-bound ligands and act as autocrine or paracrine effectors. In syndecans, this process is mediated by matrix metalloproteinases (MMPs), whereas in glypicans it results from proteolytic or lipolytic cleavage, leading to the release of the entire molecule. Shedding contributes to tumor progression by producing soluble bioactive SDC-1, which, once released into the microenvironment, enhances angiogenesis and invasiveness [25]. Recent research has shown that HS and HSPGs can move into the cell nucleus. They influence the cell cycle and growth, and they also affect gene transcription by blocking histone acetyltransferase (HAT) and disrupting the transcription machinery [26].

## 7. Serglycin

Serglycin is a distinctive proteoglycan, being the only intracellular member of this family, predominantly localized in secretory vesicles of hematopoietic and endothelial cells. Its core protein is composed of multiple serine–glycine repeats, and it is best characterized as a hybrid proteoglycan carrying both highly sulfated chains of heparan sulfate and chondroitin sulfate chains, whose composition varies among cell types. In connective tissue and mucosal mast cells, serglycin is essential for secretory granule biogenesis, as its highly sulfated heparan sulfate chains mediate electrostatic interactions with proteases and inflammatory mediators, thereby promoting their storage and stabilization [27,28]. Upon activation, mast cells release their granule contents. Although the serglycin contains the specific pentasaccharide sequence that confers anticoagulant activity, its physiological role in mast cells is likely to be local and primarily related to protease storage and regulation rather than systemic anticoagulation [5,28].

## 8. HSPG Post-Translational Modification

As mentioned before, the ECM plays a multitude of roles in tissues, from physical to biochemical support. Cells are constantly rebuilding, and the maintenance of this dynamic structure involves well-regulated synthesis, degradation, reassembly, and chemical modification. ECM is constantly remodeled by a cohort of different degradative enzymes. Among these, a peculiar class of enzymes can be influenced by post-biosynthetic modifications in the composition of HSPGs owing to the removal of specific sulfate groups. These enzymes are sulfatases that selectively remove the 6-O-sulfate groups from glucosamine in the HS chains and heparanase (HPSE), the only human enzyme capable of inducing an intrachain cut of HS, promoting the release of diffusible HS fragments. Together with shedding, the removal of specific sulfate groups by sulfatases and cleavage of HS chains are other post-biosynthetic modifications of HSPGs that modify the capability of this versatile set of molecules. Although both heparanase and sulfatases play key roles in the remodeling of HSPGs, hereafter, we will focus our discussion on HPSE, the only human enzyme known to catalyze intrachain cleavage.

## 9. HSPGs and Their Enzymatic Degradation: Heparanase

**Figure 2:**
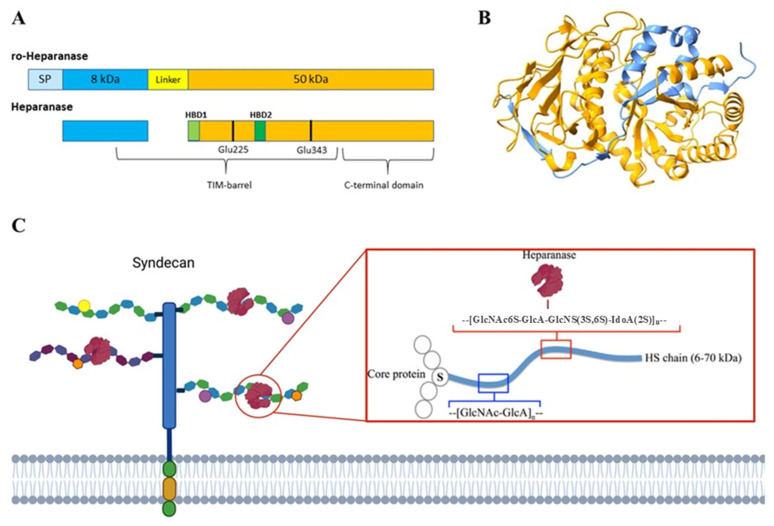
Heparanase sequence and structure. In (**A**), a diagram of human pro-heparanase and heparanase sequences is shown, while (**B**) shows the 3D crystallographic structure of human heparanase (PDB code 5E8M from the RSCB Protein Data Bank and modeled with UCSF ChimeraX 1.9 software). Light blue represents the signal peptide (SP); in blue, the 8 kDa chain; in yellow, the 6 kDa linker peptide; in orange, the 50 kDa chain; and in light green and green, HBD-1 and HBD-2, respectively. The black stick highlights two catalytic amino acid Glu residues. In (**C**) is a schematic representation of the HPSE β-endoglucuronidase activity. Image edited from [44].

Heparanase-1 (HPSE) was first isolated from the placenta and later from platelets [29]. It was cloned and characterized in the 1999s by different groups [30,31,32,33]; however, the first was the group of Vlodavsky et al. (1999) [33]. This enzyme is the only endo-β-D-glucuronidase identified in humans and specifically cleaves the β-1,4-glycosidic bond within defined trisaccharide motifs (GlcNS/GlcNAc-GlcA-GlcNS) of heparan sulfate chains that exhibit a particular sulfation pattern, thereby producing HS fragments of 5–10 kDa [34] (Figure 2). The human HPSE gene is located on chromosome 4q21.3, and by alternative splicing, two mRNAs contain the same open reading frame (ORF) [33]. Additionally, a related protein termed HPSE-2 with approximately 40% sequence similarity but one that lacks glycosidase activity and appears to function as an endogenous inhibitor of HPSE [35].

Heparanase is initially synthesized as a 543-aa pre-proenzyme (pre-proHPSE, 68 kDa) that undergoes a complex maturation process (Figure 2A). Following removal of the N-terminal signal peptide during translocation into the endoplasmic reticulum (ER), it is converted into the latent 65 kDa pro-HPSE form, which is subsequently processed in the Golgi apparatus. Glycosylation at six predicted sites is critical for its trafficking through the ER and Golgi and for its final secretion [36]. The precursor is then packaged into vesicles and released into the extracellular space, where it associates with various membrane proteins, most notably SDC-1, mannose-6-phosphate, and low-density lipoprotein receptors [36,37,38]. The resulting protein–substrate complex is internalized by endocytosis and transported to late endosomes, which fuse with lysosomes. Here, cathepsin L cleaves a 6 kDa linker peptide, generating two subunits of the mature enzyme [39].

HPSE belongs to the glycoside hydrolase family 79 (GH79), members of which typically require proton donors and nucleophilic residues for catalysis. In HPSE, Glu225 and Glu343, both within the main subunit, serve as the essential catalytic residues (Figure 2A). The mature active enzyme arises from noncovalent interactions between an 8 kDa N-terminal fragment and a 50 kDa C-terminal fragment. Structural studies of human HPSE-1 revealed that the enzyme comprises a (β/α)8 TIM barrel domain and a β-sandwich domain. The 8 kDa subunit contributes one β-sheet to the β-sandwich and provides the initial β–α–β motif of the TIM barrel [40] (Figure 2B). Within the TIM barrel lie two heparin/HS-binding domains (HBDs): HBD1 (Lys158–Asp162) at the N-terminus of the main subunit, and HBD2 (Gln270–Lys280) located in the fifth α-helix [41]. The C-terminal region of the 50 kDa subunit (amino acids 413–Ile543) is essential for completing the β-sandwich domain and is crucial for secretion, as well as for both the enzymatic and non-enzymatic functions of HPSE [42]. The active enzyme localizes to perinuclear acidic endosomes and lysosomal granules in fibroblasts, neutrophils, and tumor cells, from which it is secreted in a tightly regulated, signal-dependent manner [43].

### Different Functions of HPSE: Enzymatic and Non-Enzymatic Activities

As mentioned in the previous paragraph, HPSE exhibits a well-documented enzymatic activity that can influence the ECM environment both directly and indirectly. It also possesses a non-enzymatic function, which is not fully understood. Although numerous studies have explored the enzymatic activity of this enzyme, only a few have investigated its non-enzymatic roles. The physiological role of HSPE is linked to the degradation and turnover of cell surface HSPGs, primarily located in the perinuclear acidic endosomal and lysosomal granules of fibroblasts, neutrophils, and tumor cells [43,45]. Extracellular active HPSE not only directly impacts the architecture of the basal membrane and ECM, but also facilitates the release and diffusion of HS-linked molecules, such as growth factors and cytokines, thereby influencing cell motility, proliferation, angiogenesis, and inflammation [46]. In addition to its established enzymatic activity, HPSE has emerged as a non-enzymatic regulator of diverse cellular functions. Both pro-HPSE and the mature enzyme can engage unidentified membrane receptors, thereby triggering signaling cascades and modulating gene expression linked to multiple biological processes. These pathways, notably PI3K/Akt, Src, and p38 MAPK, promote cell adhesion, migration, and angiogenesis. Akt activation, in particular, depends on RICTOR–mTOR and is facilitated by integrins [47]. Current efforts are concentrated on identifying the receptors responsible for mediating HPSE’s non-enzymatic functions of HPSE. Notably, recent findings have demonstrated that the receptor that facilitates HPSE-induced Akt phosphorylation is situated within lipid rafts [48]. Furthermore, HPSE is not confined to intracellular vesicles. When appropriate stimuli activate protein kinase A (PKA) and kinase C (PKC) pathways, mature HPSE can be secreted through exocytosis [43]. Following lysosomal permeabilization and association with the chaperone heat shock protein 90, active HPSE can translocate to the nucleus, where it degrades nuclear HS and modulates gene expression [26,49]. HPSE modulates the expression of genes involved in glucose metabolism and inflammation in endothelial cells, influences promyeloblast differentiation, and contributes to tumorigenesis in melanoma cell lines. Notably, both the mature enzyme and the latent pro-HPSE have been detected within the nucleus. The observation that exogenously supplied pro-HPSE can translocate to the nucleus and undergo maturation has led to the hypothesis that HPSE processing may also occur in this compartment [50]. Given the nuclear localization of HSPGs, it is unsurprising that HPSE has also been found in the nucleus. Two distinct modes of gene expression regulation by HPSE have been proposed: promoting HAT activity by cleaving nuclear HS and directly interacting with DNA [51,52]. HPSE has been shown to colocalize in the nucleus with SDC-1, its principal surface receptor. Nuclear localization of SDC-1 is regulated by HPSE enzymatic activity, which is linked to the transcription of genes such as MMP-9, VEGF, and hepatocyte growth factor (HGF), thereby promoting an aggressive phenotype [51]. Notably, shed SDC-1 lacks the nuclear localization consensus sequence present in the full-length molecule, suggesting that HS ligands may supply the nuclear targeting signal. Shed SDC-1 retaining intact HS chains has been shown to complex with HGF and translocate to the nuclei of myeloma cells [26].

## 10. Heparanase in Physiology

Cellular expression of HPSE is meticulously controlled to avoid unchecked HS cleavage and negative biological outcomes. Under normal conditions, methylation silences the HPSE promoter [53] and the wild-type transcription factor p53 generally suppresses its expression in most tissues [54]. HPSE is typically expressed at low levels in all tissues, with the exception of immune cells like mast cells and leukocytes, as well as platelets, keratinocytes, heart muscle, endothelial cells, and placental trophoblasts (https://www.genecards.org/cgi-bin/carddisp.pl?gene=HPSE#expression, accessed on 15 May 2025). During regular cellular activities, HPSE expression can be increased in response to immune cell activation or viral infection, mediated by NF-kB [55,56]. Regulation of HPSE involves not only gene expression but also secretion, internalization, and activation of the enzyme. For instance, sequence analysis has identified six potential N-linked glycosylation sites, and the glycosylation pattern is crucial for HPSE secretion, although not for its enzymatic activity [36]. The activation of the enzyme is closely linked to pH levels; at neutral pH, as found in the cytoplasm and ECM, HPSE remains largely inactive. However, under acidic conditions, with optimal activity at a pH of 5–6, such as during inflammation or tumor progression, HPSE becomes active [57]. Enzymatic remodeling of HS is essential for physiological processes that depend on cell motility and growth factor bioavailability. HPSE contributes to diverse events, including embryonic development, hair follicle growth, wound repair, and angiogenesis [58]. Angiogenesis is one of the most extensively studied effects of HPSE. HPSE promotes angiogenesis during wound healing. In keratinocytes, HPSE-dependent migration facilitates tissue remodeling and repair [59]. In addition, HPSE released from degranulated platelets and immune cells facilitates the interaction of inflammatory cells with subendothelial membranes, promotes their extravasation, and contributes to blood coagulation [60].

## 11. HPSE in Pathology

Through both HS degradation and non-catalytic mechanisms, HPSE is strongly implicated in numerous pathological conditions. Its upregulation has been documented in tumors as well as in inflammatory and degenerative diseases [61]. To date, several studies have demonstrated that many HPSE regulators exist, and in turn, HPSE can activate several downstream targets [62]. Here, we focused our attention on fibrosis, cancer, and tightly interconnected inflammation.

### 11.1. HPSE in Fibrosis

Tissue fibrosis can be considered a dysregulated wound-healing process, characterized by an imbalance in extracellular matrix (ECM) homeostasis, leading to an accumulation of ECM components coupled with reduced remodeling. This mechanism exhibits heterogeneity across different parenchymal organs, and HPSE appears to be involved in various fibrotic events [63]. In the context of renal fibrosis, the excessive expression of HPSE triggered by various factors, including elevated glucose levels, advanced glycosylation end products, and albuminuria [64] or ischemia/reperfusion (I/R) injury [65], results in damage to tubular epithelial and glomerular cells. HPSE modulates the signaling pathways of pro-fibrotic factors such as fibroblast growth factor-2 (FGF-2) and transforming growth factor-beta (TGF-β) [66,67]. By influencing these signaling pathways, EMT can be controlled in renal tubular cells, which is a central event in the progression of renal fibrogenesis [68,69]. Specifically, the FGF-2 autocrine loop involves activation of the PI3K/AKT pathway, leading to the downregulation of SDC1 and upregulation of matrix metalloproteinase 9 (MMP9) [67]. The interactions between TGF-β and HPSE have been elucidated in studies showing that HPSE inhibition or strategies, such as the use of BMP-7, a TGF-β antagonist, prevent fibrotic progression and chronic pro-fibrotic damage following acute kidney injury [70,71]. In liver fibrosis, HPSE plays a complex role, contributing to both fibrogenesis and potentially limiting disease progression. According to a study on a mouse model of chronic liver fibrosis induced by carbon tetrachloride, HPSE expression increases in the early stages of liver damage and mediates the activation of HSCs via macrophage interactions, thus playing a central role in the fibrotic response of the liver. HPSE is primarily located in necroinflammatory regions, suggesting its involvement in inflammation-mediated liver injury [56]. Ongoing research is exploring various molecular targets, including HPSE, to devise potential treatments. Efforts are geared towards understanding and mitigating the pathways involving HSCs and macrophage-derived HPSE secretion to manage liver fibrosis more effectively [72]. In a recent study on idiopathic pulmonary fibrosis (IPF), it was shown that HPSE contributes to IPF progression by promoting M2 macrophage polarization via the PI3K/Akt-autophagy axis. Pharmacological inhibition of HPSE by OGT2115 attenuates lung fibrosis and M2 macrophage infiltration in vivo [73]. Overall, HPSE involvement in fibrosis is complex, acting through various mechanisms and pathways across different organ systems, underscoring the potential for targeted therapeutic strategies to mitigate fibrosis through modulation of this enzyme’s activity.

### 11.2. HPSE in Inflammation

Inflammation is a protective response to tissue injury, characterized by the recruitment of circulating immune cells to the damaged site. Heparan sulfate (HS) is central to this process, as it modulates inflammation at multiple levels: sequestering cytokines and chemokines within the extracellular matrix (ECM) [74], regulating leukocyte interactions with both the endothelium and ECM [75,76], and initiating innate immune responses through binding to Toll-like receptor 4 (TLR4) [77,78]. HPSE regulates multiple aspects of inflammation, including immune cell activation and migration, the establishment of both acute and chronic inflammatory states, cytokine and chemokine release within the ECM, and lymphangiogenesis [46]. By liberating HS-bound chemokines, HPSE generates cytokine gradients—such as IL-1β, IL-6, IL-8, IL-10, and TNF-α—that drive leukocyte recruitment, rolling, and extravasation [74,79]. Leukocyte migration across the endothelium is further shaped by HS-mediated interactions with selectins and integrins, which promote cell arrest, firm adhesion, and transendothelial migration [75,76].

HPSE activity is implicated in the functions of diverse innate immune cells, including neutrophils, macrophages, and dendritic cells. Its expression enhances pancreatic cytokines (TNF-α, IL-6) and phospho-STAT3 signaling, together with edema and neutrophil infiltration, ultimately triggering acute pancreatitis [80,81]. HPSE is secreted by neutrophils, activated T lymphocytes, platelets, and endothelial cells, facilitating immune cell extravasation by remodeling the subendothelial basement membrane and increasing vascular permeability [76,82]. For instance, in a murine model of sepsis-induced lung injury, HPSE promoted neutrophil infiltration into pulmonary microvascular endothelial cells [83]. In renal tissue, HPSE expression correlates with macrophage activation by TNF-α, thereby sustaining the chronic inflammation associated with diabetic nephropathy [84]. Moreover, soluble HS fragments generated by HPSE engage Toll-like receptors (TLRs), contributing to macrophage activation [77,78].

Recent evidence from kidney ischemia/reperfusion (I/R) injury models indicates that HS fragments released by HPSE activate macrophage and proximal tubular cell TLRs, generating a pro-inflammatory cytokine gradient that attracts and activates macrophages. HPSE also promotes macrophage polarization toward an M1 pro-inflammatory phenotype [85]. Consistently, genetic deletion of HPSE in mice results in macrophages with reduced cytokine production (TNF-α, IL-1β, IL-6, IL-10), impaired phagocytic activity, and diminished infiltrative capacity [86]. HPSE has been implicated in several inflammation-driven cancers (see “Inflammation in cancer”).

## 12. HPSE Influences the Hallmark of Cancer

**Figure 3:**
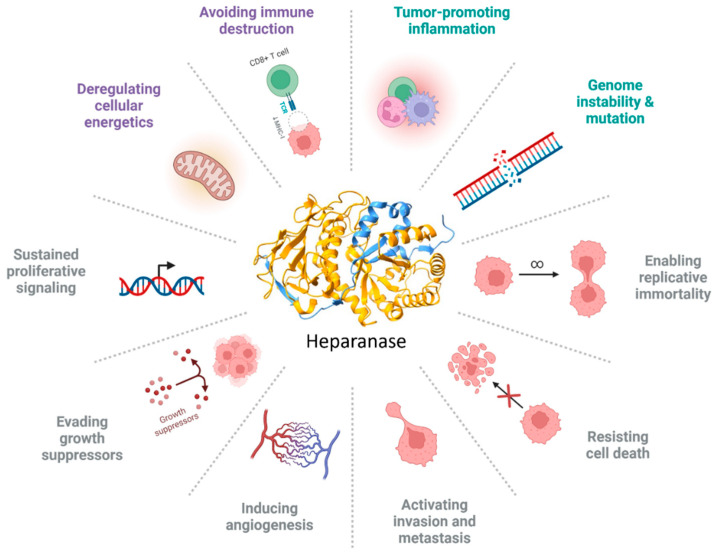
HPSE influences the hallmarks of cancer. The image summarizes all the hallmarks of cancer, and the different colors represent the following: gray, original hallmarks; purple, enabling factors; and green, emerging hallmarks. Image created by Biorender.com.

Since the first characterization by Vlodavsky, HPSE has attracted increasing attention for its potential role in cancer. This finding supports the idea that this enzyme is overexpressed in numerous human cancers and correlates with poor prognosis for patients. HPSE could be considered a “paramount” enzyme owing to its heterogeneous activity. In fact, HPSE directly or indirectly influences all the “hallmarks” of cancer that act on the ECM in the TME, adding a plasticity factor that supports tumor growth, progression, and metastasis. Its enzymatic activity leads to ECM remodeling and increases the release of HS-linked molecules (Figure 3). The less-described non-enzymatic function adds another step of complexity [87,88].

### 12.1. HPSE Influences Oncogenic, Proliferative, and Growth Signals

The role of HPSE in cancer largely derives from its capacity to modulate cellular responses across multiple levels, influencing oncogenic signaling, proliferation, and growth factor activity. Certain mutations associated with cancer initiation and progression also impact HPSE expression. Among its key functions, HPSE regulates oncogenes such as BRAF, c-Myc, and RAS, thereby promoting tumor development and progression. For instance, mutant B-Raf kinase has been shown to activate the HPSE promoter, upregulating its expression. Similarly, in vitro experiments demonstrated that HPSE mRNA levels increase in HEK293 cells transiently transfected with mutant RAS [89]. In vivo, HPSE overexpression has been correlated with RAS mutations in breast and skin cancers [90]. Moreover, human telomerase reverse transcriptase (hTERT), which sustains telomere length in many cancers, has been linked to Myc- and HPSE-driven signaling in gastric cancer. Specifically, hTERT forms a complex with c-Myc that binds and activates the HPSE promoter, thereby enhancing invasion and metastasis [91]. In addition, the HPSE/HSPG axis has been implicated in Myc oncogenic signaling in medulloblastoma [92].

**Figure 4:**
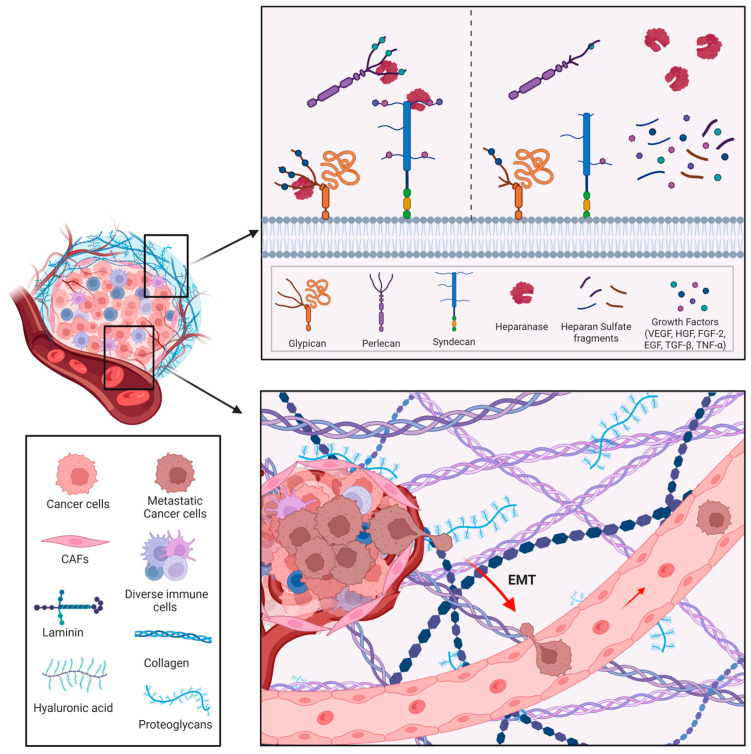
Main functions of HPSE in cancer. Heparanase can directly modify the surrounding environment (through enzymatic activity) and indirectly promote the release of many HS-linked molecules, such as growth factors, cytokines, and enzymes that sustain angiogenesis and inflammation. HPSE facilitates tumor cell migration, penetration of the basement membrane, and metastatic dissemination. Image created by Biorender.com.

Cancer cells are characterized by deregulated proliferation driven by imbalanced signaling pathways. Early studies suggested that HS binding not only stabilizes growth factors and protects them from degradation, but also serves as a reservoir for their release. Individual HSPGs appear to exert distinct roles in specific tumor contexts [93,94]. HPSE disrupts growth factor homeostasis by increasing bioavailability, as enzymatic cleavage of HS chains liberates HS-bound molecules such as FGF, HGF, and VEGF from the ECM, thereby activating signaling pathways in both tumor and stromal cells of the TME [95,96] (Figure 4).

Among these, the FGF/FGFR axis is the best characterized. All FGFs contain a globular β-trefoil domain with an HS-binding site that enables sequestration. Signal activation requires HS-mediated dimerization of FGFRs through formation of an FGF–FGFR–HS ternary complex [97]. Tissue-specific HS sulfation and epimerization further fine-tune this system. For example, 2-O-sulfated L-iduronate and N-sulfated D-glucosamine are essential for FGF2 binding, while 6-O-sulfation is required for mitogenic activity. FGF1 similarly depends on 2-O-, N-, and 6-O-sulfation for effective signaling [22]. HS fragments derived from SDC-1 cleavage by HPSE potentiate FGF2 mitogenicity [98], while nuclear translocation of SDC-1 allows shuttling of HS-binding factors, including FGF2 and HGF [26].

HGF is another HS-binding growth factor regulated by HPSE. Studies in myeloma models revealed dual roles for HPSE in HGF activity: high serum levels of HPSE, shed SDC-1, and HGF correlate with poor prognosis, reflecting functional cooperation [99]. HPSE upregulates HGF transcription and protein expression, with secreted HGF binding to HPSE-induced shed SDC-1, which amplifies signaling via the c-Met receptor. Notably, this regulation is independent of HPSE’s enzymatic activity but linked to the bioactivity of HGF mediated by shed SDC-1 [100]. Conversely, HGF itself can activate PI3K/Akt and NF-κB pathways to promote HPSE expression in gastric cancer cells, further correlating with poor prognosis [101].

HPSE is also closely tied to angiogenesis, vascular permeability, and lymphangiogenesis through modulation of VEGF. Overexpression of HPSE elevates VEGF mRNA and protein levels in multiple cancer cell models, a process mediated by p38 phosphorylation and Src activation [102].

EGFR signaling represents another pathway influenced by HPSE. EGFR overexpression is common across cancers and drives uncontrolled proliferation via downstream oncogenic cascades [103]. Heparin-binding EGF (HB-EGF), a high-affinity HS ligand, strongly activates EGFR [104]. In pancreatic ductal adenocarcinoma, high HPSE expression enhances HB-EGF activity, contributing to differentiation and lymph node metastasis [105]. Moreover, HPSE-induced SDC-1 shedding indirectly activates EGFR signaling, as soluble SDC-1 binds HB-EGF through intact HS chains, stimulating EGFR pathways and promoting chemotherapy resistance in colorectal cancer [106]. Conversely, EGF induces nucleolar localization of HPSE, which modulates DNA topoisomerase-I activity and promotes proliferation [107].

TGF-β, which functions as a tumor suppressor in early stages but becomes tumor-promoting in advanced cancers [108], also interacts with HS, which regulates its bioavailability, particularly via betaglycan [24,109]. In a non-tumorigenic model, we demonstrated that HPSE upregulation coordinates TGF-β activity to drive EMT [66], suggesting a mechanistic link between HPSE and TGF-β signaling in cancer.

### 12.2. HPSE in Cancer Angiogenesis

Angiogenesis and lymphangiogenesis are the two main mechanisms that potentiate the vascular network in cancers. They are as important as proliferation or metastatic spread, due to an adequate supply of oxygen and nutrients for the cells that compose the tumor mass. Angiogenesis and angiogenesis-associated factors are strongly associated with tumor aggressiveness [110]. HPSE is an active player involved in various aspects of neo-angiogenesis, as HSPGs are structural components of the endothelial glycocalyx of capillaries. The cleavage of HS chains promoted by HPSE contributes significantly to tumor angiogenesis, enabling endothelial cells to proliferate and migrate in response to angiogenic stimuli [88]. HPSE activity determines the release and diffusion of VEGF and FGF, two HS-binding proteins that have been shown to be potent regulators of angiogenesis in cancer [102,111,112]. A study conducted on primary breast tumors suggested that the overexpression and activity of HPSE in the TME induce the activation of VEGF and FGF signaling pathways, promoting tumor angiogenesis. MCF-7 human breast cancer cells that actively express HPSE exhibit higher angiogenesis in vivo, which is also correlated with a large tumor size [113]. A positive correlation between HPSE levels and angiogenesis has been found in a histological analysis of human colorectal cancers [114]. In addition, a recent study by Jayatilleke et al. demonstrated that in the HPSE-knockout murine mammary carcinoma model (MMTV-PyMT), angiogenesis potential was dramatically decreased in the mammary gland [115].

The mechanism of action of HPSE has been revealed in multiple myeloma. In these cells, the trimming of HS chains on SDC-1 produced by HPSE facilitates cleavage by MMP-9 (whose expression is correlated with high HPSE). Shedding of SDC-1 exposes a latent domain that promotes the interaction of VEGFR2 with α4β1 integrin, leading to the activation of the VEGF-2 receptor in both myeloma and endothelial cells. This mechanism promotes at the same time angiogenesis, invasion, and metastasis, and drives myeloma progression [25]. Another proposed HPSE-induced mechanism of angiogenesis is that HPSE activates the cyclooxygenase-2 (Cox-2)/HIF1-α pathway [116]. Indeed, in cervical cancer, it was found that HPSE in response to radiation induces not only an increase in HIF-1 but also of VEGF and FGF, promoting both radiation resistance and angiogenesis [117].

### 12.3. Invasion and Metastasis

As mentioned in the Introduction, the death of patients with breast cancer is mainly caused by metastasis. Despite significant advances in the diagnosis and treatment of cancer, metastasis is associated with more than 90% of all cancer-related deaths [118]. Metastasis is underpinned by the well-characterized cellular process of epithelial–mesenchymal transition (EMT) (Figure 4). During EMT, epithelial cells lose cell–cell junctions, apical–basal polarity, and interactions with the basement membrane, while acquiring a fibroblast-like morphology together with enhanced migratory and frequently invasive properties. EMT arises from dynamic interactions between cells and their microenvironment, which drive changes in gene expression and post-translational regulatory mechanisms, ultimately promoting phenotype switching [119]. Overexpression of ECM degradative enzymes, which impair ECM homeostasis and increase degradation potential, is important for the invasive and metastatic capacities of tumor cells. The collective expression of degradative enzymes, such as MMPs, ADAMs, ADAMTS, plasminogen activation system components, cathepsins, and HPSE in the tumor microenvironment enables invading cells to migrate inside the ECM and then disseminate into the circulation [120].

Multiple studies have shown that HPSE overexpression correlates with enhanced metastatic potential across diverse tumor types. Immunohistochemical analyses of patient samples revealed that invasive tumor regions exhibited strong HPSE positivity, whereas adjacent healthy tissues displayed no detectable signal. Conversely, inhibition of HPSE through gene silencing or selective inhibitors reduced the invasive capacity and metastatic spread of several tumor cell lines in both in vitro and in vivo models. These findings underscore the role of HPSE as a key promoter of invasion and metastasis in cancer and are summarized in Table 1.

### 12.4. HPSE in Cancer Inflammation

The tumor microenvironment (TME) is marked by chronic inflammation, leading to the description of cancers as “wounds that never heal” [137]. Within the TME, numerous immune cells play dual roles, either contributing to tumor elimination or supporting tumor growth and progression. Tumor-associated immune cells engage in cross-talk with cancer cells, driving phenotypic changes that convert them into tumor-supporting cells [138,139]. Macrophages, which constitute a substantial component of the tumor mass, are of particular relevance; in breast cancer, their abundance is regarded as a prognostic marker [140].

As mentioned before, HPSE can influence many aspects of immune cells at multiple levels (see “HPSE in inflammation”). Recent findings have indicated that HPSE is an important link between inflammation and inflammation-associated cancer. For example, HPSE has been identified as the driver of the transition from Barrett’s esophagus to esophageal adenocarcinoma. Immunohistochemical analysis showed a progressive increase in HPSE from a normal esophagus to high-grade Barrett’s esophageal carcinoma [141].

A mouse model of acute and chronic colitis demonstrated that epithelial cell–derived HPSE contributes to the modulation and sustained activation of inflammatory macrophages. In turn, these macrophages stimulate colonic epithelial cells to produce and secrete HPSE via TNF-α, with subsequent activation by cathepsin L. This persistent inflammatory loop establishes a tumor-promoting microenvironment that facilitates epithelial invasion and drives colorectal cancer (CRC) progression [142,143].

Studies on chronic gastritis induced by Helicobacter pylori (considered the major risk factor for gastric cancer) have suggested that HPSE is upregulated and is involved in the early stages of gastric cancer. Similarly, in colorectal cancer, the role of HPSE is mainly associated with the recruitment of macrophages, generating a vicious cycle (driven by the NF-kB and p38-MAPK signaling pathways) that sustains chronic inflammation, supporting the development and progression of gastric cancer [144].

HPSE is not only able to drive cancer growth and progression but can also support immune evasion, an emerging role associated with tumor-associated macrophages (TAMs). It is noteworthy that HPSE plays an important role in the activation and function of macrophages, which in turn protects the tumor in two different ways: (1) by the expression of the human leukocyte antigen (HLA), which in turn prevents the activation of NK cells and some T cells; (2) by the release of chemokines, T regulatory cells that inhibit the activity of CD4+ and CD8+ T cells [145].

Given its involvement in multiple aspects of immune cell function, HPSE may also exert beneficial effects in cancer therapy. Recent findings by Caruana et al. demonstrated that HPSE plays a critical role in chimeric antigen receptor (CAR)-T cell therapy. Specifically, HPSE expression in long-term ex vivo-expanded, tumor-specific CAR-T cells enhances their ability to degrade the ECM, thereby improving antitumor efficacy [146].

### 12.5. HPSE Role in Cell Death Evasion

Cell death is considered one of the hallmarks of cancer. Indeed, during progression, cancer cells must acquire the capability to escape apoptosis and undergo physiologically programmed cell death. This is due to the inhibition of apoptosis promoted by the upregulation of anti-apoptotic signals or by the deregulation of pro-apoptotic signals [147]. In this context, HPSE plays an anti-apoptotic role in both enzymatic and non-enzymatic activities. In breast cancer, the release of FGF promoted by HPSE is correlated with inhibition of apoptosis and prolonged tumor survival [113]. In addition, an RNA-seq experiment in HPSE-overexpressing MCF-7 cells identified the regulation of apoptosis as a potential pathway associated with cell viability after 5-fluorouracil treatment [148].

Autophagy is another well-described cellular mechanism that directly contributes to cancer cell survival and leads to chemoresistance. This evolutionarily conserved catabolic pathway contributes to cellular homeostasis by degrading damaged cellular components [149,150]. Recent data suggest that the expression of HPSE promotes autophagy through a reduction in mTOR1, the key regulator of autophagy, by promoting tumor growth and chemoresistance [151]. In addition, new evidence has indicated that both active and inactive HPSE modulate TFEB-mediated autophagy in gastric cancer cells [152].

In hepatocellular carcinoma (HCC) cells, high HPSE expression was shown to induce necroptosis in adjacent microvascular endothelial cells (MVECs), thereby promoting trans-endothelial migration through the HPSE/SDC-1/TNF-α and p38 MAPK pathways. HPSE knockdown reversed necroptosis and reduced TNF-α levels, whereas HPSE overexpression upregulated SDC-1 and TNF-α, exacerbating necroptosis [130].

## 13. Conclusions

Until recently, proteoglycans were primarily seen as complex structural molecules within the ECM and mainly recognized for their role as functional components of amorphous substances. However, recent experimental findings have revealed their capacity not only to regulate the structural and mechanical organization of the matrix, but also to influence the key signaling cascades that dictate cellular behavior. This review focuses on HSPGs, which exhibit the greatest variability in sequence and sulfation within their GAG chains, making them the primary binding sites for matrix molecules, growth factors, and cytokines. HSPGs are crucial for maintaining tissue homeostasis and in the onset and progression of various diseases through their interactions with FGF, TGFβ, VEGF, PDGF, and cell membrane receptors. HSPGs are intricately regulated by HPSE, the sole endoglycosidase that catalyzes the cleavage of HS side chains, leading to remodeling of the extracellular matrix and basement membranes and facilitating the release of various bound molecules such as growth factors, cytokines, and enzymes. As has been extensively discussed, this enzyme is implicated in numerous pathological conditions, including fibrosis, inflammation, and tumors. Although several molecules have been tested as potential inhibitors of this enzyme, no agent has been approved yet for application in the clinical treatment of these diseases. It is hoped that in the near future, new drugs will be developed to block HPSE activity, thereby restoring ECM homeostasis and reversing these pathological conditions.

## Figures and Tables

**Table 1:** HPSE expression is involved in invasion and metastasis in various tumors.

Tumor Type	Observation	Reference
Breast cancer	Analyses of fifty-one primary breast tumors showed that HPSE expression is significantly correlated with sentinel node metastasis. HPSE-positive tumors (90%) showed a reduced level of HS deposition.	[121]
Breast cancer	Serum levels of MMP-9 and HPSE are elevated in breast cancer patients and show a positive correlation with histological grade, lymph node status, and lymphovascular invasion.	[122]
Breast cancer	miR-1258 has been shown to suppress breast cancer brain metastasis in vitro by targeting the 3′-untranslated region of HPSE, thereby inhibiting its expression and activity.	[123]
Cervical cancer	HPSE expression in cervical cancer patients correlates with tumor size and clinical stage via immunohistochemistry analyses. HPSE-overexpressing cervical cancer cells increased proliferation in vitro and tumor growth in vivo.	[124]
Colorectal cancer	Knockdown of HPSE in different colorectal cancer cell lines inhibited invasion and liver metastasis in vitro and in vivo. RNA-seq showed alteration in invasion and metastasis-related genes.	[125]
Gastric cancer	HPSE mRNA expression significantly correlates with late-stage, large-size, lymph nodal metastasis via in situ hybridization of primary gastric carcinomas.	[126]
Gastric cancer	miR-299-3p targets the 3′-UTR of HPSE mRNA, regulating its expression. Similarly, miR-1258 demonstrated a reduction in HPSE protein and gene expression, reducing invasion and metastasis in gastric cancer cells in vitro.	[127,128]
Head and neck squamous cell carcinomas (HNSCCs)	In situ hybridization analyses have demonstrated that HPSE expression is associated with lymph node metastasis in HNSCC biopsies. Furthermore, both in vitro and in vivo studies confirmed a correlation between HPSE expression and reduced disease-free and overall survival.	[129]
Hepatocellular cancer	Highly expressed HPSE induces necroptosis of the adjacent microvascular endothelial cells (MVECs), activating the HPSE/SDC-1/TNF-α axis and p38 MAPK pathway that promote intrahepatic metastasis.	[130]
Melanoma	Immunohistochemistry analyses demonstrated that high levels of HPSE were associated with late-stage melanoma patients.	[131]
Multiple myeloma	HPSE drives multiple myeloma metastasis and progression, enhancing Fibronectin and Vimentin partially due to the activation of the ERK pathway in vitro and in vivo.	[132]
Ovarian cancer	Elevated serum Cathepsin L, HPSE, and MMP-9 levels are correlated with malignant invasion and progression in ovarian cancer.	[133]
Pancreatic cancer	Pancreatic cancer cells transfected with a full-length HPSE construct exhibited increased invasiveness in invasion chamber assays. In situ hybridization further showed that HPSE expression in pancreatic cancer correlates with clinicopathologic parameters. Kaplan–Meier survival analysis using the log-rank test revealed that HPSE expression in early-stage tumors was associated with reduced patient survival.	[134]
Prostate cancer	In situ hybridization demonstrated that HPSE mRNA expression in prostate carcinomas was significantly correlated with tumor differentiation and tumor stage.	[135]
Prostate cancer	In vitro experiments showed that HPSE expression influences EMT and stemness marker expression in two different prostate cancer cell lines.	[136]

## Data Availability

No new data were created or analyzed in this study.

## References

[1] Karamanos N.K., Theocharis A.D., Piperigkou Z., Manou D., Passi A., Skandalis S.S., Vynios D.H., Orian-Rousseau V., Ricard-Blum S., Schmelzer C.E.H. (2021). A Guide to the Composition and Functions of the Extracellular Matrix. FEBS J..

[2] Naba A. (2023). Ten Years of Extracellular Matrix Proteomics: Accomplishments, Challenges, and Future Perspectives. Mol. Cell. Proteom..

[3] Mecham R.P. (2012). Overview of Extracellular Matrix. Curr. Protoc. Cell Biol..

[4] Hynes R.O., Naba A. (2012). Overview of the Matrisome--An Inventory of Extracellular Matrix Constituents and Functions. Cold Spring Harb. Perspect. Biol..

[5] Karamanos N.K., Piperigkou Z., Theocharis A.D., Watanabe H., Franchi M., Baud S., Brézillon S., Götte M., Passi A., Vigetti D. (2018). Proteoglycan Chemical Diversity Drives Multifunctional Cell Regulation and Therapeutics. Chem. Rev..

[6] Iozzo R.V., Schaefer L. (2015). Proteoglycan Form and Function: A Comprehensive Nomenclature of Proteoglycans. Matrix Biol..

[7] Sarrazin S., Lamanna W.C., Esko J.D. (2011). Heparan Sulfate Proteoglycans. Cold Spring Harb. Perspect. Biol..

[8] Vlodavsky I., Barash U., Nguyen H.M., Yang S.-M., Ilan N. (2021). Biology of the Heparanase–Heparan Sulfate Axis and Its Role in Disease Pathogenesis. Semin. Thromb. Hemost..

[9] Hassan N., Greve B., Espinoza-Sánchez N.A., Götte M. (2021). Cell-Surface Heparan Sulfate Proteoglycans as Multifunctional Integrators of Signaling in Cancer. Cell. Signal..

[10] Ravikumar M., Smith R.A.A., Nurcombe V., Cool S.M. (2020). Heparan Sulfate Proteoglycans: Key Mediators of Stem Cell Function. Front. Cell Dev. Biol..

[11] Marques C., Reis C.A., Vivès R.R., Magalhães A. (2021). Heparan Sulfate Biosynthesis and Sulfation Profiles as Modulators of Cancer Signalling and Progression. Front. Oncol..

[12] Annaval T., Wild R., Crétinon Y., Sadir R., Vivès R.R., Lortat-Jacob H. (2020). Heparan Sulfate Proteoglycans Biosynthesis and Post Synthesis Mechanisms Combine Few Enzymes and Few Core Proteins to Generate Extensive Structural and Functional Diversity. Molecules.

[13] Multhaupt H.A.B., Couchman J.R. (2012). Heparan Sulfate Biosynthesis: Methods for Investigation of the Heparanosome. J. Histochem. Cytochem..

[14] Lamanna W.C., Kalus I., Padva M., Baldwin R.J., Merry C.L.R., Dierks T. (2007). The Heparanome—The Enigma of Encoding and Decoding Heparan Sulfate Sulfation. J. Biotechnol..

[15] Ori A., Wilkinson M.C., Fernig D.G. (2008). The Heparanome and Regulation of Cell Function: Structures, Functions and Challenges. Front. Biosci..

[16] Farach-Carson M.C., Warren C.R., Harrington D.A., Carson D.D. (2014). Border Patrol: Insights into the Unique Role of Perlecan/Heparan Sulfate Proteoglycan 2 at Cell and Tissue Borders. Matrix Biol..

[17] Gubbiotti M.A., Neill T., Iozzo R.V. (2017). A Current View of Perlecan in Physiology and Pathology: A Mosaic of Functions. Matrix Biol..

[18] Daniels M.P. (2012). The Role of Agrin in Synaptic Development, Plasticity and Signaling in the Central Nervous System. Neurochem. Int..

[19] Heljasvaara R., Aikio M., Ruotsalainen H., Pihlajaniemi T. (2017). Collagen XVIII in Tissue Homeostasis and Dysregulation—Lessons Learned from Model Organisms and Human Patients. Matrix Biol..

[20] Bishop J.R., Schuksz M., Esko J.D. (2007). Heparan Sulphate Proteoglycans Fine-Tune Mammalian Physiology. Nature.

[21] Afratis N.A., Nikitovic D., Multhaupt H.A.B., Theocharis A.D., Couchman J.R., Karamanos N.K. (2017). Syndecans—Key Regulators of Cell Signaling and Biological Functions. FEBS J..

[22] Mohammadi M., Olsen S.K., Ibrahimi O.A. (2005). Structural Basis for Fibroblast Growth Factor Receptor Activation. Cytokine Growth Factor Rev..

[23] Filmus J. (2023). Glypicans, 35 Years Later. Proteoglycan Res..

[24] Villarreal M.M., Kim S.K., Barron L., Kodali R., Baardsnes J., Hinck C.S., Krzysiak T.C., Henen M.A., Pakhomova O., Mendoza V. (2016). Binding Properties of the Transforming Growth Factor-β Coreceptor Betaglycan: Proposed Mechanism for Potentiation of Receptor Complex Assembly and Signaling. Biochemistry.

[25] Jung O., Trapp-Stamborski V., Purushothaman A., Jin H., Wang H., Sanderson R.D., Rapraeger A.C. (2016). Heparanase-Induced Shedding of Syndecan-1/CD138 in Myeloma and Endothelial Cells Activates VEGFR2 and an Invasive Phenotype: Prevention by Novel Synstatins. Oncogenesis.

[26] Stewart M.D., Ramani V.C., Sanderson R.D. (2015). Shed Syndecan-1 Translocates to the Nucleus of Cells Delivering Growth Factors and Inhibiting Histone Acetylation. J. Biol. Chem..

[27] Kolset S.O., Tveit H. (2008). Serglycin—Structure and Biology. Cell. Mol. Life Sci..

[28] Mulloy B., Lever R., Page C.P. (2017). Mast cell glycosaminoglycans. Glycoconj. J..

[29] Vlodavsky I., Kayal Y., Hilwi M., Soboh S., Sanderson R.D., Ilan N. (2023). Heparanase—A Single Protein with Multiple Enzymatic and Nonenzymatic Functions. Proteoglycan Res..

[30] Hulett M.D., Freeman C., Hamdorf B.J., Baker R.T., Harris M.J., Parish C.R. (1999). Cloning of Mammalian Heparanase, an Important Enzyme in Tumor Invasion and Metastasis. Nat. Med..

[31] Kussie P.H., Hulmes J.D., Ludwig D.L., Patel S., Navarro E.C., Seddon A.P., Giorgio N.A., Bohlen P. (1999). Cloning and Functional Expression of a Human Heparanase Gene. Biochem. Biophys. Res. Commun..

[32] Toyoshima M., Nakajima M. (1999). Human Heparanase. J. Biol. Chem..

[33] Vlodavsky I., Friedmann Y., Elkin M., Aingorn H., Atzmon R., Ishai-Michaeli R., Bitan M., Pappo O., Peretz T., Michal I. (1999). Mammalian Heparanase: Gene Cloning, Expression and Function in Tumor Progression and Metastasis. Nat. Med..

[34] Peterson S.B., Liu J. (2013). Multi-Faceted Substrate Specificity of Heparanase. Matrix Biol..

[35] McKenzie E., Tyson K., Stamps A., Smith P., Turner P., Barry R., Hircock M., Patel S., Barry E., Stubberfield C. (2000). Cloning and Expression Profiling of Hpa2, a Novel Mammalian Heparanase Family Member. Biochem. Biophys. Res. Commun..

[36] Simizu S., Ishida K., Wierzba M.K., Osada H. (2004). Secretion of Heparanase Protein Is Regulated by Glycosylation in Human Tumor Cell Lines. J. Biol. Chem..

[37] Levy-Adam F., Miao H.-Q., Heinrikson R.L., Vlodavsky I., Ilan N. (2003). Heterodimer Formation Is Essential for Heparanase Enzymatic Activity. Biochem. Biophys. Res. Commun..

[38] Ben-Zaken O., Shafat I., Gingis-Velitski S., Bangio H., Kelson I.K., Alergand T., Amor Y., Maya R.B.-Y., Vlodavsky I., Ilan N. (2008). Low and High Affinity Receptors Mediate Cellular Uptake of Heparanase. Int. J. Biochem. Cell Biol..

[39] Abboud-Jarrous G., Atzmon R., Peretz T., Palermo C., Gadea B.B., Joyce J.A., Vlodavsky I. (2008). Cathepsin L Is Responsible for Processing and Activation of Proheparanase through Multiple Cleavages of a Linker Segment. J. Biol. Chem..

[40] Wu L., Viola C.M., Brzozowski A.M., Davies G.J. (2015). Structural Characterization of Human Heparanase Reveals Insights into Substrate Recognition. Nat. Struct. Mol. Biol..

[41] Levy-Adam F., Abboud-Jarrous G., Guerrini M., Beccati D., Vlodavsky I., Ilan N. (2005). Identification and Characterization of Heparin/Heparan Sulfate Binding Domains of the Endoglycosidase Heparanase. J. Biol. Chem..

[42] Fux L., Feibish N., Cohen-Kaplan V., Gingis-Velitski S., Feld S., Geffen C., Vlodavsky I., Ilan N. (2009). Structure-Function Approach Identifies a COOH-Terminal Domain That Mediates Heparanase Signaling. Cancer Res..

[43] Shafat I., Vlodavsky I., Ilan N. (2006). Characterization of Mechanisms Involved in Secretion of Active Heparanase. J. Biol. Chem..

[44] Rivara S., Milazzo F.M., Giannini G. (2016). Heparanase: A Rainbow Pharmacological Target Associated to Multiple Pathologies Including Rare Diseases. Future Med. Chem..

[45] Goldshmidt O., Nadav L., Aingorn H., Irit C., Feinstein N., Ilan N., Zamir E., Geiger B., Vlodavsky I., Katz B.Z. (2002). Human Heparanase Is Localized within Lysosomes in a Stable Form. Exp. Cell Res..

[46] Masola V., Bellin G., Gambaro G., Onisto M. (2018). Heparanase: A Multitasking Protein Involved in Extracellular Matrix (ECM) Remodeling and Intracellular Events. Cells.

[47] Riaz A., Ilan N., Vlodavsky I., Li J.-P., Johansson S. (2013). Characterization of Heparanase-Induced Phosphatidylinositol 3-Kinase-AKT Activation and Its Integrin Dependence. J. Biol. Chem..

[48] Ben-Zaken O., Gingis-Velitski S., Vlodavsky I., Ilan N. (2007). Heparanase Induces Akt Phosphorylation via a Lipid Raft Receptor. Biochem. Biophys. Res. Commun..

[49] Nobuhisa T., Naomoto Y., Okawa T., Takaoka M., Gunduz M., Motoki T., Nagatsuka H., Tsujigiwa H., Shirakawa Y., Yamatsuji T. (2007). Translocation of Heparanase into Nucleus Results in Cell Differentiation. Cancer Sci..

[50] Schubert S.Y., Ilan N., Shushy M., Ben-Izhak O., Vlodavsky I., Goldshmidt O. (2004). Human Heparanase Nuclear Localization and Enzymatic Activity. Lab. Investig..

[51] Purushothaman A., Hurst D.R., Pisano C., Mizumoto S., Sugahara K., Sanderson R.D. (2011). Heparanase-Mediated Loss of Nuclear Syndecan-1 Enhances Histone Acetyltransferase (HAT) Activity to Promote Expression of Genes That Drive an Aggressive Tumor Phenotype. J. Biol. Chem..

[52] Yang Y., Gorzelanny C., Bauer A.T., Halter N., Komljenovic D., Bäuerle T., Borsig L., Roblek M., Schneider S.W. (2015). Nuclear Heparanase-1 Activity Suppresses Melanoma Progression via Its DNA-Binding Affinity. Oncogene.

[53] Shteper P.J., Zcharia E., Ashhab Y., Peretz T., Vlodavsky I., Ben-Yehuda D. (2003). Role of Promoter Methylation in Regulation of the Mammalian Heparanase Gene. Oncogene.

[54] Baraz L., Haupt Y., Elkin M., Peretz T., Vlodavsky I. (2006). Tumor Suppressor P53 Regulates Heparanase Gene Expression. Oncogene.

[55] Agelidis A.M., Hadigal S.R., Jaishankar D., Shukla D. (2017). Viral Activation of Heparanase Drives Pathogenesis of Herpes Simplex Virus-1. Cell Rep..

[56] Secchi M.F., Crescenzi M., Masola V., Russo F.P., Floreani A., Onisto M. (2017). Heparanase and Macrophage Interplay in the Onset of Liver Fibrosis. Sci. Rep..

[57] Nagarajan H., Vetrivel U. (2018). Demystifying the pH Dependent Conformational Changes of Human Heparanase Pertaining to Structure–Function Relationships: An in Silico Approach. J. Comput. Aided Mol. Des..

[58] Nasser N.J. (2008). Heparanase Involvement in Physiology and Disease. Cell. Mol. Life Sci..

[59] Zcharia E., Zilka R., Yaar A., Yacoby-Zeevi O., Zetser A., Metzger S., Sarid R., Naggi A., Casu B., Ilan N. (2005). Heparanase Accelerates Wound Angiogenesis and Wound Healing in Mouse and Rat Models. FASEB J..

[60] Nadir Y. (2014). Heparanase and Coagulation–New Insights. Rambam Maimonides Med. J..

[61] Secchi M.F., Masola V., Zaza G., Lupo A., Gambaro G., Onisto M. (2015). Recent Data Concerning Heparanase: Focus on Fibrosis, Inflammation and Cancer. Biomol. Concepts.

[62] Mayfosh A.J., Nguyen T.K., Hulett M.D. (2021). The Heparanase Regulatory Network in Health and Disease. Int. J. Mol. Sci..

[63] Masola V., Gambaro G., Onisto M., Vlodavsky I., Sanderson R.D., Ilan N. (2020). Impact of Heparanse on Organ Fibrosis. Heparanase.

[64] Masola V., Gambaro G., Tibaldi E., Onisto M., Abaterusso C., Lupo A. (2011). Regulation of Heparanase by Albumin and Advanced Glycation End Products in Proximal Tubular Cells. Biochim. Biophys. Acta (BBA) Mol. Cell Res..

[65] Masola V., Zaza G., Gambaro G., Onisto M., Bellin G., Vischini G., Khamaysi I., Hassan A., Hamoud S., Nativ O. (2016). Heparanase: A Potential New Factor Involved in the Renal Epithelial Mesenchymal Transition (EMT) Induced by Ischemia/Reperfusion (I/R) Injury. PLoS ONE.

[66] Masola V., Zaza G., Secchi M.F., Gambaro G., Lupo A., Onisto M. (2014). Heparanase Is a Key Player in Renal Fibrosis by Regulating TGF-β Expression and Activity. Biochim. Biophys. Acta (BBA) Mol. Cell Res..

[67] Masola V., Gambaro G., Tibaldi E., Brunati A.M., Gastaldello A., D’Angelo A., Onisto M., Lupo A. (2012). Heparanase and Syndecan-1 Interplay Orchestrates Fibroblast Growth Factor-2-Induced Epithelial-Mesenchymal Transition in Renal Tubular Cells. J. Biol. Chem..

[68] Masola V., Zaza G., Onisto M., Lupo A., Gambaro G. (2015). Impact of Heparanase on Renal Fibrosis. J. Transl. Med..

[69] Abassi Z., Hamoud S., Hassan A., Khamaysi I., Nativ O., Heyman S.N., Muhammad R.S., Ilan N., Singh P., Hammond E. (2017). Involvement of Heparanase in the Pathogenesis of Acute Kidney Injury: Nephroprotective Effect of PG545. Oncotarget.

[70] Kim S., Jeong C.-H., Song S.H., Um J.E., Kim H.S., Yun J.S., Han D., Cho E.S., Nam B.Y., Yook J.I. (2020). Micellized Protein Transduction Domain-Bone Morphogenetic Protein-7 Efficiently Blocks Renal Fibrosis Via Inhibition of Transforming Growth Factor-Beta–Mediated Epithelial–Mesenchymal Transition. Front. Pharmacol..

[71] Masola V., Bellin G., Vischini G., Dall’Olmo L., Granata S., Gambaro G., Lupo A., Onisto M., Zaza G. (2018). Inhibition of Heparanase Protects against Chronic Kidney Dysfunction Following Ischemia/Reperfusion Injury. Oncotarget.

[72] Zhang C.-Y., Yuan W.-G., He P., Lei J.-H., Wang C.-X. (2016). Liver Fibrosis and Hepatic Stellate Cells: Etiology, Pathological Hallmarks and Therapeutic Targets. World J. Gastroenterol..

[73] He L., Lv Q., Luo J., Guo Y.-D., Sun H., Zong M., Fan L.-Y. (2025). Heparanase Inhibition Mitigates Bleomycin-Induced Pulmonary Fibrosis in Mice by Reducing M2 Macrophage Polarization. Immunol. Lett..

[74] Xie M., Li J. (2019). Heparan Sulfate Proteoglycan—A Common Receptor for Diverse Cytokines. Cell. Signal..

[75] Higashi N., Irimura T., Nakajima M., Vlodavsky I., Sanderson R.D., Ilan N. (2020). Heparanase Is Involved in Leukocyte Migration. Heparanase.

[76] Masola V., Greco N., Gambaro G., Franchi M., Onisto M. (2022). Heparanase as Active Player in Endothelial Glycocalyx Remodeling. Matrix Biol. Plus.

[77] Goodall K.J., Poon I.K.H., Phipps S., Hulett M.D. (2014). Soluble Heparan Sulfate Fragments Generated by Heparanase Trigger the Release of Pro-Inflammatory Cytokines through TLR-4. PLoS ONE.

[78] Elkin M., Vlodavsky I., Sanderson R.D., Ilan N. (2020). Role of Heparanase in Macrophage Activation. Heparanase.

[79] Collins L.E., Troeberg L. (2018). Heparan Sulfate as a Regulator of Inflammation and Immunity. J. Leukoc. Biol..

[80] Khamaysi I., Singh P., Nasser S., Awad H., Chowers Y., Sabo E., Hammond E., Gralnek I., Minkov I., Noseda A. (2017). The Role of Heparanase in the Pathogenesis of Acute Pancreatitis: A Potential Therapeutic Target. Sci. Rep..

[81] Hamo-Giladi D.B., Fokra A., Sabo E., Kabala A., Minkov I., Hamoud S., Hadad S., Abassi Z., Khamaysi I. (2024). Involvement of Heparanase in the Pathogenesis of Acute Pancreatitis: Implication of Novel Therapeutic Approaches. J. Cell. Mol. Med..

[82] Goldberg R., Meirovitz A., Hirshoren N., Bulvik R., Binder A., Rubinstein A.M., Elkin M. (2013). Versatile Role of Heparanase in Inflammation. Matrix Biol..

[83] Schmidt E.P., Yang Y., Janssen W.J., Gandjeva A., Perez M.J., Barthel L., Zemans R.L., Bowman J.C., Koyanagi D.E., Yunt Z.X. (2012). The Pulmonary Endothelial Glycocalyx Regulates Neutrophil Adhesion and Lung Injury during Experimental Sepsis. Nat. Med..

[84] Goldberg R., Rubinstein A.M., Gil N., Hermano E., Li J.-P., van der Vlag J., Atzmon R., Meirovitz A., Elkin M. (2014). Role of Heparanase-Driven Inflammatory Cascade in Pathogenesis of Diabetic Nephropathy. Diabetes.

[85] Masola V., Zaza G., Bellin G., Dall’Olmo L., Granata S., Vischini G., Francesca Secchi M., Lupo A., Gambaro G., Onisto M. (2018). Heparanase Regulates the M1 Polarization of Renal Macrophages and Their Crosstalk with Renal Epithelial Tubular Cells after Ischemia/Reperfusion Injury. FASEB J..

[86] Waterman M., Ben-Izhak O., Eliakim R., Groisman G., Vlodavsky I., Ilan N. (2007). Heparanase Upregulation by Colonic Epithelium in Inflammatory Bowel Disease. Mod. Pathol..

[87] Jayatilleke K.M., Hulett M.D. (2020). Heparanase and the Hallmarks of Cancer. J. Transl. Med..

[88] Masola V., Greco N., Gambaro G., Franchi M., Onisto M., Kovalszky I., Franchi M., Alaniz L.D. (2022). Heparanase: A Paramount Enzyme for Cancer Initiation, Progression, and Metastasis. The Extracellular Matrix and the Tumor Microenvironment.

[89] Rao G., Liu D., Xing M., Tauler J., Prinz R.A., Xu X. (2010). Induction of Heparanase-1 Expression by Mutant B-Raf Kinase: Role of GA Binding Protein in Heparanase-1 Promoter Activation. Neoplasia.

[90] Boyango I., Barash U., Naroditsky I., Li J.-P., Hammond E., Ilan N., Vlodavsky I. (2014). Heparanase Cooperates with *Ras.* to Drive Breast and Skin Tumorigenesis. Cancer Res..

[91] Tang B., Xie R., Qin Y., Xiao Y.-F., Yong X., Zheng L., Dong H., Yang S.-M. (2016). Human Telomerase Reverse Transcriptase (hTERT) Promotes Gastric Cancer Invasion through Cooperating with c-Myc to Upregulate Heparanase Expression. Oncotarget.

[92] Ridgway L.D., Wetzel M.D., Marchetti D. (2011). Heparanase Modulates Shh and Wnt3a Signaling in Human Medulloblastoma Cells. Exp. Ther. Med..

[93] Gallagher J. (2015). Fell–Muir Lecture: Heparan Sulphate and the Art of Cell Regulation: A Polymer Chain Conducts the Protein Orchestra. Int. J. Exp. Path.

[94] Vallet S.D., Berthollier C., Ricard-Blum S. (2022). The Glycosaminoglycan Interactome 2.0. Am. J. Physiol. Cell Physiol..

[95] Masola V., Zaza G., Gambaro G., Franchi M., Onisto M. (2020). Role of Heparanase in Tumor Progression: Molecular Aspects and Therapeutic Options. Semin. Cancer Biol..

[96] Vlodavsky I., Singh P., Boyango I., Gutter-Kapon L., Elkin M., Sanderson R.D., Ilan N. (2016). Heparanase: From Basic Research to Therapeutic Applications in Cancer and Inflammation. Drug Resist. Updates.

[97] Goetz R., Mohammadi M. (2013). Exploring Mechanisms of FGF Signalling through the Lens of Structural Biology. Nat. Rev. Mol. Cell Biol..

[98] Kato M., Wang H., Kainulainen V., Fitzgerald M.L., Ledbetter S., Ornitz D.M., Bernfield M. (1998). Physiological Degradation Converts the Soluble Syndecan-1 Ectodomain from an Inhibitor to a Potent Activator of FGF-2. Nat. Med..

[99] Alexandrakis M.G., Passam F.H., Sfiridaki A., Kandidaki E., Roussou P., Kyriakou D.S. (2003). Elevated Serum Concentration of Hepatocyte Growth Factor in Patients with Multiple Myeloma: Correlation with Markers of Disease Activity. Am. J. Hematol..

[100] Ramani V.C., Yang Y., Ren Y., Nan L., Sanderson R.D. (2011). Heparanase Plays a Dual Role in Driving Hepatocyte Growth Factor (HGF) Signaling by Enhancing HGF Expression and Activity. J. Biol. Chem..

[101] Hao N.-B., Tang B., Wang G.-Z., Xie R., Hu C.-J., Wang S.-M., Wu Y.-Y., Liu E., Xie X., Yang S.-M. (2015). Hepatocyte Growth Factor (HGF) Upregulates Heparanase Expression via the PI3K/Akt/NF-κB Signaling Pathway for Gastric Cancer Metastasis. Cancer Lett..

[102] Zetser A., Bashenko Y., Edovitsky E., Levy-Adam F., Vlodavsky I., Ilan N. (2006). Heparanase Induces Vascular Endothelial Growth Factor Expression: Correlation with P38 Phosphorylation Levels and Src Activation. Cancer Res..

[103] Wee P., Wang Z. (2017). Epidermal Growth Factor Receptor Cell Proliferation Signaling Pathways. Cancers.

[104] Normanno N., De Luca A., Bianco C., Strizzi L., Mancino M., Maiello M.R., Carotenuto A., De Feo G., Caponigro F., Salomon D.S. (2006). Epidermal Growth Factor Receptor (EGFR) Signaling in Cancer. Gene.

[105] Hoffmann A.-C., Mori R., Vallbohmer D., Brabender J., Drebber U., Baldus S.E., Klein E., Azuma M., Metzger R., Hoffmann C. (2008). High Expression of Heparanase Is Significantly Associated with Dedifferentiation and Lymph Node Metastasis in Patients with Pancreatic Ductal Adenocarcinomas and Correlated to PDGFA and Via HIF1a to HB-EGF and bFGF. J. Gastrointest. Surg..

[106] Wang X., Zuo D., Chen Y., Li W., Liu R., He Y., Ren L., Zhou L., Deng T., Wang X. (2014). Shed Syndecan-1 Is Involved in Chemotherapy Resistance via the EGFR Pathway in Colorectal Cancer. Br. J. Cancer.

[107] Zhang L., Sullivan P., Suyama J., Marchetti D. (2010). Epidermal Growth Factor–Induced Heparanase Nucleolar Localization Augments DNA Topoisomerase I Activity in Brain Metastatic Breast Cancer. Mol. Cancer Res..

[108] Principe D.R., Doll J.A., Bauer J., Jung B., Munshi H.G., Bartholin L., Pasche B., Lee C., Grippo P.J. (2014). TGF-: Duality of Function Between Tumor Prevention and Carcinogenesis. JNCI J. Natl. Cancer Inst..

[109] Troilo H., Steer R., Collins R.F., Kielty C.M., Baldock C. (2016). Independent Multimerization of Latent TGFβ Binding Protein-1 Stabilized by Cross-Linking and Enhanced by Heparan Sulfate. Sci. Rep..

[110] Nishida N., Yano H., Nishida T., Kamura T., Kojiro M. (2006). Angiogenesis in Cancer. Vasc. Health Risk Manag..

[111] Marchetti D., Reiland J., Kempf D., Roy M., Denkins Y. (2006). FGF2 Binding, Signaling and Angiogenesis Are Modulated by Heparanase in Metastatic Melanoma Cells. Melanoma Res..

[112] Liu G., Chen T., Ding Z., Wang Y., Wei Y., Wei X. (2021). Inhibition of FGF-FGFR and VEGF-VEGFR Signalling in Cancer Treatment. Cell Prolif..

[113] Cohen I., Pappo O., Elkin M., San T., Bar-Shavit R., Hazan R., Peretz T., Vlodavsky I., Abramovitch R. (2006). Heparanase Promotes Growth, Angiogenesis and Survival of Primary Breast Tumors. Int. J. Cancer.

[114] Sato T., Yamaguchi A., Goi T., Hirono Y., Takeuchi K., Katayama K., Matsukawa S. (2004). Heparanase Expression in Human Colorectal Cancer and Its Relationship to Tumor Angiogenesis, Hematogenous Metastasis, and Prognosis. J. Surg. Oncol..

[115] Jayatilleke K.M., Duivenvoorden H.M., Ryan G.F., Parker B.S., Hulett M.D. (2023). Investigating the Role of Heparanase in Breast Cancer Development Utilising the MMTV-PyMT Murine Model of Mammary Carcinoma. Cancers.

[116] Naomoto Y., Gunduz M., Takaoka M., Okawa T., Gunduz E., Nobuhisa T., Kobayashi M., Shirakawa Y., Yamatsuji T., Sonoda R. (2007). Heparanase Promotes Angiogenesis through Cox-2 and HIF1α. Med. Hypotheses.

[117] Li J., Meng X., Hu J., Zhang Y., Dang Y., Wei L., Shi M. (2017). Heparanase Promotes Radiation Resistance of Cervical Cancer by Upregulating Hypoxia Inducible Factor. Am. J. Cancer Res..

[118] Steeg P.S. (2006). Tumor Metastasis: Mechanistic Insights and Clinical Challenges. Nat. Med..

[119] Yang J., Antin P., Berx G., Blanpain C., Brabletz T., Bronner M., Campbell K., Cano A., Casanova J., Christofori G. (2020). Guidelines and Definitions for Research on Epithelial–Mesenchymal Transition. Nat. Rev. Mol. Cell Biol..

[120] Piperigkou Z., Kyriakopoulou K., Koutsakis C., Mastronikolis S., Karamanos N.K. (2021). Key Matrix Remodeling Enzymes: Functions and Targeting in Cancer. Cancers.

[121] Maxhimer J.B., Quiros R.M., Stewart R., Dowlatshahi K., Gattuso P., Fan M., Prinz R.A., Xu X. (2002). Heparanase-1 Expression Is Associated with the Metastatic Potential of Breast Cancer. Surgery.

[122] Tang D., Piao Y., Zhao S., Mu X., Li S., Ma W., Song Y., Wang J., Zhao W., Zhang Q. (2014). Expression and Correlation of Matrix Metalloproteinase-9 and Heparanase in Patients with Breast Cancer. Med. Oncol..

[123] Zhang L., Sullivan P.S., Goodman J.C., Gunaratne P.H., Marchetti D. (2011). MicroRNA-1258 Suppresses Breast Cancer Brain Metastasis by Targeting Heparanase. Cancer Res..

[124] Zeng C., Ke Z.-F., Luo W.-R., Yao Y.-H., Hu X.-R., Jie W., Yin J.-B., Sun S.-J. (2013). Heparanase Overexpression Participates in Tumor Growth of Cervical Cancer in Vitro and in Vivo. Med. Oncol..

[125] Liu X., Zhou Z., Li W., Zhang S., Li J., Zhou M.-J., Song J.-W. (2019). Heparanase Promotes Tumor Growth and Liver Metastasis of Colorectal Cancer Cells by Activating the P38/MMP1 Axis. Front. Oncol..

[126] Tang W., Nakamura Y., Tsujimoto M., Sato M., Wang X., Kurozumi K., Nakahara M., Nakao K., Nakamura M., Mori I. (2002). Heparanase: A Key Enzyme in Invasion and Metastasis of Gastric Carcinoma. Mod. Pathol..

[127] Shi J., Chen P., Sun J., Song Y., Ma B., Gao P., Chen X., Wang Z. (2017). MicroRNA-1258: An Invasion and Metastasis Regulator That Targets Heparanase in Gastric Cancer. Oncol. Lett..

[128] Zhou X., Hu M., Ge Z. (2019). Tumor-suppressive miR-299-3p Inhibits Gastric Cancer Cell Invasion by Targeting Heparanase. Mol. Med. Rep..

[129] Beckhove P., Helmke B.M., Ziouta Y., Bucur M., Dörner W., Mogler C., Dyckhoff G., Herold-Mende C. (2005). Heparanase Expression at the Invasion Front of Human Head and Neck Cancers and Correlation with Poor Prognosis. Clin. Cancer Res..

[130] Chen X., Cheng B., Dai D., Wu Y., Feng Z., Tong C., Wang X., Zhao J. (2021). Heparanase Induces Necroptosis of Microvascular Endothelial Cells to Promote the Metastasis of Hepatocellular Carcinoma. Cell Death Discov..

[131] Vornicova O., Boyango I., Feld S., Naroditsky I., Kazarin O., Zohar Y., Tiram Y., Ilan N., Ben-Izhak O., Vlodavsky I. (2016). The Prognostic Significance of Heparanase Expression in Metastatic Melanoma. Oncotarget.

[132] Li J., Pan Q., Rowan P.D., Trotter T.N., Peker D., Regal K.M., Javed A., Suva L.J., Yang Y. (2016). Heparanase Promotes Myeloma Progression by Inducing Mesenchymal Features and Motility of Myeloma Cells. Oncotarget.

[133] Zhang W., Yang H.-C., Wang Q., Yang Z.-J., Chen H., Wang S.-M., Pan Z.-M., Tang B.-J., Li Q.Q., Li L. (2011). Clinical Value of Combined Detection of Serum Matrix Metalloproteinase-9, Heparanase, and Cathepsin for Determining Ovarian Cancer Invasion and Metastasis. Anticancer Res..

[134] Kim A.W., Xu X., Hollinger E.F., Gattuso P., Godellas C.V., A Prinz R. (2002). Human Heparanase-1 Gene Expression in Pancreatic Adenocarcinoma. J. Gastrointest. Surg..

[135] Stadlmann S., Moser P.L., Pollheimer J., Steiner P., Krugmann J., Dirnhofer S., Mikuz G., Margreiter R., Amberger A. (2003). Heparanase-1 Gene Expression in Normal, Hyperplastic and Neoplastic Prostatic Tissue. Eur. J. Cancer.

[136] Masola V., Franchi M., Zaza G., Atsina F.M., Gambaro G., Onisto M. (2022). Heparanase Regulates EMT and Cancer Stem Cell Properties in Prostate Tumors. Front. Oncol..

[137] Singel K.L., Segal B.H. (2016). Neutrophils in the Tumor Microenvironment: Trying to Heal the Wound That Cannot Heal. Immunol. Rev..

[138] Dehne N., Mora J., Namgaladze D., Weigert A., Brüne B. (2017). Cancer Cell and Macrophage Cross-Talk in the Tumor Microenvironment. Curr. Opin. Pharmacol..

[139] Marzagalli M., Ebelt N.D., Manuel E.R. (2019). Unraveling the Crosstalk between Melanoma and Immune Cells in the Tumor Microenvironment. Semin. Cancer Biol..

[140] Medrek C., Pontén F., Jirström K., Leandersson K. (2012). The Presence of Tumor Associated Macrophages in Tumor Stroma as a Prognostic Marker for Breast Cancer Patients. BMC Cancer.

[141] Brun R., Naroditsky I., Waterman M., Ben-Izhak O., Groisman G., Ilan N., Vlodavsky I. (2009). Heparanase Expression by Barrett’s Epithelium and during Esophageal Carcinoma Progression. Mod. Pathol..

[142] Menzel K., Hausmann M., Obermeier F., Schreiter K., Dunger N., Bataille F., Falk W., Scholmerich J., Herfarth H., Rogler G. (2006). Cathepsins B, L and D in Inflammatory Bowel Disease Macrophages and Potential Therapeutic Effects of Cathepsin Inhibition in Vivo. Clin. Exp. Immunol..

[143] Lerner I., Hermano E., Zcharia E., Rodkin D., Bulvik R., Doviner V., Rubinstein A.M., Ishai-Michaeli R., Atzmon R., Sherman Y. (2011). Heparanase Powers a Chronic Inflammatory Circuit That Promotes Colitis-Associated Tumorigenesis in Mice. J. Clin. Investig..

[144] Tang L., Tang B., Lei Y., Yang M., Wang S., Hu S., Xie Z., Liu Y., Vlodavsky I., Yang S. (2021). Helicobacter Pylori-Induced Heparanase Promotes H. Pylori Colonization and Gastritis. Front. Immunol..

[145] Mantovani A., Allavena P., Marchesi F., Garlanda C. (2022). Macrophages as Tools and Targets in Cancer Therapy. Nat. Rev. Drug Discov..

[146] Caruana I., Savoldo B., Hoyos V., Weber G., Liu H., Kim E.S., Ittmann M.M., Marchetti D., Dotti G. (2015). Heparanase Promotes Tumor Infiltration and Antitumor Activity of CAR-Redirected T Lymphocytes. Nat. Med..

[147] Fernald K., Kurokawa M. (2013). Evading Apoptosis in Cancer. Trends Cell Biol..

[148] Zahavi T., Salmon-Divon M., Salgado R., Elkin M., Hermano E., Rubinstein A.M., Francis P.A., Di Leo A., Viale G., de Azambuja E. (2021). Heparanase: A Potential Marker of Worse Prognosis in Estrogen Receptor-Positive Breast Cancer. npj Breast Cancer.

[149] Levy J.M.M., Towers C.G., Thorburn A. (2017). Targeting Autophagy in Cancer. Nat. Rev. Cancer.

[150] Mulcahy Levy J.M., Thorburn A. (2020). Autophagy in Cancer: Moving from Understanding Mechanism to Improving Therapy Responses in Patients. Cell Death Differ..

[151] Shteingauz A., Boyango I., Naroditsky I., Hammond E., Gruber M., Doweck I., Ilan N., Vlodavsky I. (2015). Heparanase Enhances Tumor Growth and Chemoresistance by Promoting Autophagy. Cancer Res..

[152] Yang M., Tang B., Wang S., Tang L., Wen D., Vlodavsky I., Yang S.-M. (2022). Non-Enzymatic Heparanase Enhances Gastric Tumor Proliferation via TFEB-Dependent Autophagy. Oncogenesis.

